# Supplementary material for: A set of multi-entry identification keys to African frugivorous flies (Diptera, Tephritidae)
Source: Zookeys. 2014 Jul 24;(428):97–108. doi: 10.3897/zookeys.428.7366 (PMC4143993; doi:10.3897/zookeys.428.7366)
Supplement: Supplementary material 6 — Key to Ceratitis [file zookeys-428-097-s006.zip › SF6_ZooKeys_key to Ceratitis/key/SF6_ZooKeys_key to Ceratitis/Media/Html/C. acicularis.htm]

Ceratitis (Pterandrus) acicularis (Munro)


***Ceratitis*** **(*Pterandrus*) *acicularis*
(Munro)**

*Pterandrus
acicularis* Munro,
1969: 420.

Body length:
5.09 (4.80-5.30) mm; wing length: 5.68 (5.25-6.15) mm.

Male

Head: Antenna yellow. First flagellomere 2-3 times as long
as pedicel. Arista with moderately long rays; ventral rays somewhat shorter and
sparser than dorsal rays, especially basally. Frons yellow; with short
scattered setulae largely of same color as frons. Frontal setae well developed,
occasionally less so. Face yellowish white. Genal seta and setulae dark, well
developed.

Thorax: Postpronotal lobe white,
without spot. Scutal pattern, ground color pale gray, with streaks and darker
markings but without distinct spots or clearly defined stripes, except
prescutellar white markings, separate but with pale area in between. Scapular
setae dark. Scutellum grayish white, basally without spots, apically with three
separate black spots, extending to basal half. Anepisternum completely pale,
ventral margin narrowly dark brown; setulae pale except few dark setulae
usually present along ventral margin.

Legs:
Yellow except where otherwise noted; setation typical for subgenus, mainly
dark. Foreleg: femur anteriorly mainly brownish, only basal and ventral margin
yellow, posteriorly largely brown, no distinct bush, only dispersed setulae;
ventral setae black. Midleg: femur anteriorly largely brown, distal end pale;
ventrally with dark dispersed setulae, distally somewhat denser but without
distinct feathering; tibia broadened; largely brown, with black feathering dorsally
along distal 0.9 and ventrally along distal 0.8, anterior part covered by short
dark setulae. Hindleg: femur brown on basal 0.8; at apical 0.25 with longer
setulae dorsally and ventrally.

Wing: Bands
brownish. Interruption between marginal and discal bands near vein R_1_
clear and complete; discal band sometimes partly interrupted in discal cell;
cubital band free; medial band absent; crossvein R-M opposite middle of discal
cell. Apex of vein R_1_ distal to level of crossvein R-M. Crossvein
DM-Cu straight.

Abdomen:
Mostly Yellow. Tergites 2 and 4 largely pale gray. Tergite 3 with brownish
black transverse band along posterior half. Tergite 5 along anterior margin
with isolated brownish patches, posterior margin narrowly black. Male epandrium
in lateral view with posterior lobe of lateral surstylus straight and elongate.

Female

As male
except following characters: Legs without feathering, yellow, except femora
yellowish orange; forefemur posteroventrally with dark pilosity. Discal band
not interrupted. Tergite 5 largely pale yellow across posterior half. Oviscape
as long as, or longer than, preabdomen. Aculeus at least eight times as long as
wide; tip with distinct apical indentation and lateral margin slightly sinuous.

(Description
after De Meyer & Freidberg, 2006)
